# Novel Bioactive Antimicrobial Lignin Containing Coatings on Titanium Obtained by Electrophoretic Deposition

**DOI:** 10.3390/ijms150712294

**Published:** 2014-07-11

**Authors:** Sanja Erakovic, Ana Jankovic, Gary C. P. Tsui, Chak-Yin Tang, Vesna Miskovic-Stankovic, Tatjana Stevanovic

**Affiliations:** 1 Faculty of Technology and Metallurgy, University of Belgrade, Karnegijeva 4, Belgrade 11000, Serbia; E-Mails: serakovic@tmf.bg.ac.rs (S.E.); ajankovic@tmf.bg.ac.rs (A.J.); vesna@tmf.bg.ac.rs (V.M.-S.); 2 Department of Industrial and Systems Engineering, Faculty of Engineering, the Hong Kong Polytechnic University, AG711 Chung Sze Yuen Building, Hung Hom, Kowloon, Hong Kong, China; E-Mails: gary.c.p.tsui@polyu.edu.hk (G.C.P.T.); cy.tang@polyu.edu.hk (C.-Y.T.); 3 Département des Sciences du Bois et de la Forêt, Faculté de Foresterie, de Géographie et de Géomatique, Université Laval, 2425 rue de la Terrasse, Pavillon Gene-H.-Kruger, Québec G1V 0A6, QC, Canada

**Keywords:** organosolv lignin, silver, hydroxyapatite, electrophoretic deposition, titanium, bioactivity, SBF, cytotoxicity, antimicrobial activity

## Abstract

Hydroxyapatite (HAP) is the most suitable biocompatible material for bone implant coatings; its brittleness, however, is a major obstacle, and the reason why research focuses on creating composites with biopolymers. Organosolv lignin (Lig) is used for the production of composite coatings, and these composites were examined in this study. Titanium substrate is a key biomedical material due to its well-known properties, but infections of the implantation site still impose a serious threat. One approach to prevent infection is to improve antimicrobial properties of the coating material. Silver doped hydroxyapatite (Ag/HAP) and HAP coatings on titanium were obtained by an electrophoretic deposition method in order to control deposited coating mass and morphology by varying applied voltage and deposition time. The effect of lignin on microstructure, morphology and thermal behavior of biocomposite coatings was investigated. The results showed that higher lignin concentrations protect the HAP lattice during sintering, improving coating stability. The corrosion stability was evaluated in simulated body fluid (SBF) at 37 °C. Newly formed plate-shaped carbonate-HAP was detected, indicating enhanced bioactive performance. The antimicrobial efficiency of Ag/HAP/Lig was confirmed by its higher reduction of bacteria *Staphylococcus aureus* TL (*S. aureus* TL) than of HAP/Lig coating. Cytotoxicity assay revealed that both coatings can be classified as non-toxic against healthy immunocompetent peripheral blood mononuclear cells (PBMC).

## 1. Introduction

Traditional metallic implants are irreplaceable in repairing damaged bone tissue, but the greatest concern is their gradual electrochemical degradation [1]. Bone fractures are one of the most common forms of injury along with bone diseases and that is why metallic implants with bioactive and biocompatible-coated material are often used in order to enhance the bone healing process [2].

Synthetic hydroxyapatite (HAP, Ca_10_(PO_4_)_6_(OH)_2_) has long been known as one of the best coating materials for metallic implants due to its biocompatible, osteoconductive and osteoinductive properties [3]. Besides controlling the stoichiometry of synthetic HAP, the control of crystallinity, porosity, particle shape, surface area and agglomeration characteristics are of great interest [4,5].

Materials implanted in the human body face an environment that is extremely delicate, but at the same time hostile. The implants face a severe corrosive environment that includes blood and body fluid composed of several constituents (water, sodium, chlorine, proteins, plasma, and amino acids) along with mucin in the case of saliva. The noncompatible metal ions released by the implants into the body are found to cause allergic and toxic reactions. In order to minimize direct contact between metal and body fluids, and to limit the release of undesired metallic ions in the body, biocompatible and bioactive coatings, on the metallic substrate, such as HAP, are suggested by many researchers [6,7,8].

Titanium and its alloys have become the material of choice for long-term implant application for their favorable corrosion resistance as well as their low toxicity, biocompatibility and good mechanical properties, such as high strength, durability, and light weight [9,10,11,12]. Hence, a good combination of the biocompatibility of hydroxyapatite and the excellent mechanical properties of titanium is considered a promising approach to fabricate more suitable bone implants. The concept of coating titanium implant surfaces with HAP combines the mechanical benefits of metal alloys with the biocompatibility of HAP [13].

Post-operative infections are the result of bacterial adhesion to the implant surface and subsequent biofilm formation at the implantation site [14]. In order to stop bacterial infection, it is crucial to inhibit bacterial adhesion since biofilm can be very resistant to immune response and antibiotics [15]. The antimicrobial activity of silver and silver ions has been known for a very long time; additionally, silver cation does not develop bacterial resistance and at the same time shows low toxicity to human cells [16,17]. Therefore, the possibility to prevent the bone implant infections by using antimicrobial properties of Ag has generated great interest in the development of silver doped HAP coatings [18].

There are various methods to deposit ceramic coatings on metal surfaces, such as plasma spraying, sputtering, pulsed laser deposition, sol-gel, electrophoresis, and electrodeposition [19]. Among these, electrophoretic deposition (EPD) emerges as a method of choice due to its simple setup and formation of uniform coatings, even on substrates of complex shape [20,21,22,23]. Other advantages are that EPD represents an inexpensive electrochemical technique that can be carried out at room temperature with the possibility of coating thickness and morphology, well controlled by adjusting deposition parameters. The necessary condition that enables successful EPD is a stable suspension/sol, where the particles have a high zeta potential while the ionic conductivity of the suspension is kept at a low value [24].

In recent years, research has strived to improve the biocomposite HAP/polymer coatings and other functional properties of the implant, such as good adhesion properties, chemical stability, bioactivity, biocompatibility and antimicrobial properties [25]. Biodegradable natural or synthetic polymers are used in the development of new biocomposite coatings. Use of the polymer dictates that thermal treatment of the composite material to be performed at lower sintering temperatures [26]. Development of biocomposite HAP/biopolymer coatings is especially important for applications in medicine, specifically transplantational surgery, because its mechanical properties are most similar in characteristics to natural bone tissue [27]. Other characteristics of biocomposite coating that make it biocompatible are non-toxicity, corrosion stability and controlled biodegradibility, as well as elastic modulus, therefore suitable for specific biomedical applications. The use of natural biopolymers, such as polysaccharides—alginate, chitosan/chitin and hyaluronic acid; proteins—collagen and silk; as well as different biofibers—lignin and cellulose, offers the advantage of improving the adhesion of bioceramic coating by decreasing its brittleness [27]. Significant interest and investigations have been focused on the fabrication of HAP/lignin composites and coatings [28,29,30,31,32].

Based on recent research developments organosolv lignin emerged as a suitable candidate for composite hydroxyapatite/natural polymer coatings. Lignin (Lig) is a complex natural polyphenolic polymer connected with a variety of chemical bonds [33]. Lignin possesses antioxidant and antimicrobial properties; therefore, its incorporation is interesting in medical applications due to of its thermal stability and biocompatibility in different materials [28,30,31]. Incorporation of lignin is interesting in medical applications because it led to improved thermal stability, hydrophilicity, biocompatibility and biodegradability of composites [28,30]. As complex natural polymer networks composed primarily of phenolic moieties, lignins have a wide variety of chemical bonds [32,33]. Among the functional groups present in lignin, the most reactive chemical sites are phenolic hydroxyl groups [34]. Other major chemical functional groups in lignins include methoxyl, carbonyl and carboxyl groups, varying on the plant origin and the applied pulping processes [34]. Organosolv lignins are being examined because they show significantly improved solubility and thermal properties compared to sulfite or kraft lignins [29]. The organosolv processes are convenient because of the greater simplicity of the chemical recovery system, since only the solvent has to be recovered by rectification of the black liquors [35]. In contrast to Kraft and sulfite lignin, Alcell (organosolv) lignin derives from a process using ethanol as the only pulping chemical and consists of low molecular weight phenol fragments with enhanced hydrophobicity [33]. Hence, this organosolv lignin, in its purified form, possesses a chemical structure different to that of native lignin. Biocomposite hydroxyapatite/lignin as a 3D scaffold was first studied by Mansur *et al.* [30] where organosolv lignin polymer was used. Pan *et al.* [36] examined the conditions of solubility and physico-chemical properties of extracted organosolv lignin. Excellent solubility of lignin, more than 90%, is obtained from the solution, which contained more than 65% of ethanol. For the same reason, the HAP/Lig coatings on titanium precipitated from ethanol suspensions.

Our assumption was that organosolv lignin will help in providing a more stable composite in order to accommodate a better interconnected porous structure to improve osteogenesis. It is our belief that lignin can help diminish the severe cracking of HAP coatings, as reported by Wang *et al.* [37] for the composite with chitosan.

Therefore, supported by the newest research developments in the field of different polymers, we have focused our efforts on developing a novel hydroxyapatite/lignin (HAP/Lig) and silver/hydroxyapatite/lignin (Ag/HAP/Lig) biocomposite coatings on titanium by EPD technique, mimicking the structure and properties of natural bone [32,38,39,40]. Based on available data, our idea was to employ organosolv lignin (Alcell) as the most suitable component for HAP composite that would provide improved porosity structure to prompt osteogenesis.

## 2. Electrophoretic Deposition of Hydroxyapatite/Lignin (HAP/Lig) and Silver/Hydroxyapatite/Lignin (Ag/HAP/Lig) Coatings on Titanium

### 2.1. Materials

#### 2.1.1. Alcell Lignin

Organosolv Alcell lignin (Repap Enterprises Inc., Stamford, CT, USA) was extracted from a mixture of North American hardwoods (maple, birch, and poplar) by an organosolv process using a mixture of ethanol–water (50/50). After ethanol removal through flashing, lignin is simply precipitated upon water addition (change of solvent). The Klason lignin and acid soluble lignin contents were determined to be 96.5% and 0.4% of Alcell™ lignin while total carbohydrates represented 0.32%, nitrogen content was 0.14% and the ash content was 0.1%. The phenolic hydroxyl content of Alcell™ lignin was determined to be 2.4 mmol/g as compared to the theoretical content of syringic acid phenolic content of 5.1 mmol/g. Very low content of 0.78 mmol/g of carboxylic acid functional groups has been determined in Alcell™ lignin. The weight average molar mass was determined to range between 6820 and 11,000 Da, while the number average molecular mass ranged between 1320 and 1900 Da for the Alcell™ lignin [35]. The lignin powder was used as received and without further purification.

#### 2.1.2. Synthesis of Nanosized Hydroxyapatite and Silver Doped Hydroxyapatite Powders

A nanosized hydroxyapatite powder was obtained using a modified chemical precipitation method, by the reaction of calcium oxide (obtained by calcination of CaCO_3_ for 5 h at 1000 °C in air) with phosphoric acid added drop-wise to reach the pH value 7.4–7.6, described elsewhere [41,42]. A modified chemical precipitation method was also employed for preparing silver/hydroxyapatite (Ag/HAP, Ca_9.95_Ag_0.05_(PO_4_)_6_(OH)_2_) powder. Briefly, a stoichiometric amount of the resulting calcium oxide was mixed and stirred in distilled water for 10 min; afterward, AgNO_3_ solution was added to the suspension, yielding a final concentration of silver ion of 0.4 ± 0.1 *wt* % in Ag/HAP nanosized powder. When the total necessary quantity of phosphoric acid was introduced, the pH reached a value of 7.4–7.6. The obtained HAP and Ag/HAP suspensions were preheated to (94 ± 1) °C for 30 min and stirred for another half an hour. After sedimentation, the upper clear solution layer was decanted. The suspensions were then spray-dried at (120 ± 5) °C into granulated powders [32,39].

#### 2.1.3. Particle Size Distribution and Zeta (ζ) Potential

The particle size distribution (PSD) and the value of zeta (ζ) potential are very important factors to establish the stability of the colloidal systems and possibilities to perform successful cathaphoretic deposition. The PSD measurement of HAP/Lig and Ag/HAP/Lig with 1 *wt* % Lig suspensions was made by using dynamic whatlight scattering technique. The HAP/Lig and Ag/HAP/Lig particle size distribution and Zeta (ζ) potential were determined using a Zeta-Sizer Nano ZS with 633 nm He–Ne laser (Malvern Inc., Malvern, UK). The instrument can measure particle size ranging from 0.6 nm to 6 μm. The obtained average particle size is around 363.0 nm, for HAP/Lig suspension, and 207.3 nm, for Ag/HAP/Lig suspension [32,39]. It can be assumed for both suspensions that the larger particles are agglomerates of smaller ones, since transmission electron microscopy images of the same powder, previously published [42], demonstrated that HAP particles are nano-sized in the range of 50–100 nm. Based on PSD intensities these prepared suspensions are agglomerates of the smaller ones. Also, it was noticed that pure HAP suspension had much higher average particle size value (1500 nm) compared to HAP/Lig suspension [32]. Therefore, it can be concluded that lignin decreases agglomeration of HAP nanoparticles.

The ζ*-*potential is a measure of the strength of interactions between colloid particles, and hence it relates to colloid solution stability. A biomaterial’s ζ-potential indicates its electric surface properties; bioceramic particles must be electrically charged for electrophoretic deposition on metal substrates [38,39]. It is well known that a cathaphoretic deposition assumes positive ζ*-*potential of particle surface during the whole process, which can certainly be achieved at pH value lower than 4. High positive values ζ*-*potential of HAP/Lig and Ag/HAP/Lig suspensions of 28 and 29 mV, respectively, indicate positively charged particle surfaces of HAP/Lig and Ag/HAP/Lig thus enabling the attraction of particles by negatively charged cathode–titanium plate and therefore successful electrophoretic deposition of coatings.

#### 2.1.4. Titanium Surface Pretreatment

Titanium by Aldrich (foil, thickness 0.25 mm, purity 99.7%) was used as a substrate for electrophoretic deposition of HAP/Lig (65 × 5 mm for surface analysis, 10 × 5 mm for cell based assay) and Ag/HAP/Lig (20 mm × 10 mm for surface analysis, 40 × 20 mm for impedance measurements and 10 × 5 mm for cell based assay) coatings. Prior to deposition, standard mechanical pretreatment of metal plates was employed. Grit emery paper was used to polish Ti plates, followed by wet polishing with 0.3 μm alumina. After polishing, plates were degreased in acetone and then in ethanol for 15 min each in ultrasonic bath.

#### 2.1.5. Electrophoretic Deposition of HAP/Lig and Ag/HAP/Lig Coatings on Titanium

Electrophoretic deposition of HAP/Lig and Ag/HAP/Lig coatings on titanium was performed from ethanol suspensions, previously described [32,38,39,40]. Briefly, 100 mL of absolute ethanol suspensions contained 1.0000 g of nanosized HAP or 1.0034 g of nanosized Ag/HAP, which would bring the Ag concentration to 0.4 ± 0.1 *wt* % in the final suspension. Lignin concentrations were varied (0.5–10) *wt* % of Alcell lignin powder in overall suspension volume. Prior to electrodeposition, the HAP/Lig and Ag/HAP/Lig suspensions were ultrasonically treated for 30 min to obtain a homogeneous particle distribution. A three-electrode cell arrangement was used for cathodic electrodeposition. The working electrode was titanium plate and the counter electrodes were two platinum panels, placed parallel to the working electrode at a distance of 1.5 cm. The deposition parameters, applied voltage and deposition time significantly influence the coating morphology and thickness. Therefore, the impact of EPD voltage and deposition time on the mass of HAP/Lig and Ag/HAP/Lig coatings deposited on Ti from ethanol suspensions were analyzed. The process was optimized by varying EPD voltage from 50 to 100 V at different deposition times of 30 s to 5 min [32]. Increase in deposition time up to 5 min at constant voltage of 60 V enhances the mass of HAP/Lig and Ag/HAP/Lig coatings since more particles are reaching the cathode. It was observed that there is a linear increase in the mass of coatings with increasing deposition time up to 3 min, and then for a longer period of time up to 5 min the mass of the coatings very slowly increases. Extending the deposition time amplifies the number of particles that reach the cathode, but the fact is also that current consumption is equal to the strength of the current dissolution of the coating after a long period of deposition, therefore the further mass increases very slowly. The increase in applied voltage up to 100 V at constant deposition time of 5 min increases the rate of particle migration and mass of HAP/Lig and Ag/HAP/Lig coatings. However, the mass of Ag/HAP/Lig coating at a constant deposition time of 5 min indicates that the mass initially increases with increasing voltage up to 60 V, whereupon it can be seen that the mass of the coating is constant with further voltage increase.

It was noticed that at lower voltages and deposition times thinner coatings were obtained, while at higher voltages and prolonged times greater coating thickness and cracks were observed (as determined by scanning electron microscopy (SEM) analysis, data not shown) [32]. Increasing the deposition time, greater amounts of hydrogen evolved from the cathode resulting in the appearance of pores in deposited coated material.

The optimal ratio of coating mass and porosity in bioceramic composite HAP coatings, with and without silver, on titanium was achieved at constant voltage of 60 V for 45 s, therefore for all further analyses of composite coatings were obtained from ethanol suspensions using these deposition conditions [32].

Electrodeposited HAP/Lig and Ag/HAP/Lig coatings were air dried for 24 h at room temperature. Afterwards, the coatings were sintered at 900 °C for 30 min in argon atmosphere, with the initial heating rate of 16 °C/min. Before sintering, the oxygen was eliminated from coatings in argon atmosphere at 200 °C for 45 min [32,38,39].

### 2.2. Methods of Testing

#### 2.2.1. Scanning Electron Microscopy (SEM)

The morphology of the electrodeposited coatings was analyzed by scanning electron microscopy, using JEOL JSM-5800 instrument (JEOL Ltd., Tokyo, Japan).

#### 2.2.2. X-ray Diffraction (XRD)

Philips PW 1051 Powder Diffractometer (Royal Philips, Amsterdam, The Netherlands) with Ni filtered Cu *K*_α_ radiation (λ = 1.5418 Å) was employed to determine phase composition of electrodeposited coatings. X-ray diffraction intensity was measured using scan-step technique (2θ = 8–80°), scanning step width of 0.05° and exposure time of 50 s per step. The phase analysis was done by commercially available computer program EVA V.9.0 implementing PDF-2 database.

The mean crystallite domain size (*D*_p_) was calculated from the half height width (β_1/2_) of the X-ray diffraction (XRD) reflection of (002) plane (at 2θ ≈ 26°), using the Scherer Equation (1):

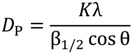
(1)
where λ is the wavelength of the X-ray radiation, *K* is the shape coefficient equal to 0.9 and θ is the diffraction angle. No microstrain corrections were taken into account.

#### 2.2.3. Attenuated Total Reflection Fourier Transform Infrared Spectroscopy (ATR-FTIR)

Attenuated total reflection Fourier transform infrared measurements were carried out with a Spectrum™ 400 PerkinElmer Infrared Spectrometer (PerkinElmer, Waltham, MA, USA) in the wavenumber 600–4000 cm^–^^1^ in order to investigate the functional groups present in electrodeposited coatings. The measurements were performed at a resolution of 4 cm^−^^1^ and 64 scans were acquired.

#### 2.2.4. X-ray Photoelectron Spectroscopy (XPS)

The surface chemical analysis of electrodeposited coatings was performed by Axis-Ultra instrument by Kratos spectrometer (XPS, Kratos Analytical Ltd., Manchester, UK) using non- and monochromatized Al *K*_α_ radiation (1486.6 eV) operating at 300 W and pass energy of 160 eV for the low-resolution survey scans and a hemispherical analyzer. XPS was employed over a wide range of 0–1100 eV under a high vacuum of 5 × 10^−10^ Torr. The take-off angle of the emitted photoelectrons was adjusted to 30° with respect to the surface normal. The XPS spectra were background subtracted, using the non-linear, iterative Shirley method. Peak fitting was processed using CasaXPS software (John T. Grant, Fairborn, OH, USA), which automatically and iteratively minimizes the difference between the experimental spectrum and the calculated envelope by varying the parameters supplied in a first guess. The fitting procedure allowed signals to be evaluated by determining the peak position, height, width and Gaussian/Lorentzian ratio.

#### 2.2.5. *In Vitro* Bioactivity Test

The *in vitro* bioactivity of Ag/HAP/Lig coatings was tested by immersion in 10 mL simulated body fluid (SBF) solution, which was refreshed every 24 h. After 7 days the samples were taken out, rinsed in deionized water and then air-dried before characterization by SEM, XRD and attenuated total reflection Fourier transform infrared spectroscopy (ATR-FTIR) analysis.

#### 2.2.6. Electrochemical Impedance Spectroscopy (EIS)

For corrosion stability estimation, electrochemical impedance spectroscopy measurements were employed on the electrodeposited Ag/HAP/Lig coating (exposed to SBF solution at 37 °C for 14 days). A standard three-electrode setup arrangement was used for the EIS experiments. The working electrode (coating or bare titanium) had testing surface area of 1 cm^2^. The counter electrode was a platinum mesh and the reference electrode was saturated calomel electrode (SCE). The impedance data were collected at the open-circuit potential using Reference 600™ Potentiostat/Galvanostat/ZRA (Gamry Instruments, Inc., Warminister, PA, USA). All of the experiments were done at the frequency range of 300 kHz to 10 mHz using 5 mV amplitude of sinusoidal voltage. Custom software Gamry Instruments Echem Analyst fitting program, version 5.50 (Gamry Instruments, Warminster, PA, USA), was used for analyzing impedance spectra.

#### 2.2.7. Inductively Coupled Plasma Spectrometry (ICP)

The release of silver was monitored in SBF solution, mimicking human body conditions. In order to investigate the silver release kinetics Ag/HAP/Lig coatings were immersed in 5 mL of SBF at 37 °C for 10 days. All experiments were performed at least in duplicate and an average of three measurements was taken for each sample. The SBF was changed periodically each day during 10 days of immersion, and the concentration of silver ion in solution was determined by inductively coupled plasma spectrometry (ICP–AES), using Varian SpectrAA 55B (Agilent Technologies, Santa Clara, CA, USA).

#### 2.2.8. Nanoindentation

The elastic modulus and hardness of sintered electrodeposited coatings were analyzed by Nanoindentation test. The test was conducted using a Hysitron Triboscope Nanomechanical System (Hysitron Inc., Minneapolis, MN, USA) with an *in situ* imaging mode and a Berkovich indenter at an applied force of 10,000 μN, while the loading and unloading rates were kept at 2000 μN/s without holding time. Eight to twelve indentations at various desired locations on the coating were made for each sample.

#### 2.2.9. 3-(4,5-Dimethylthiazol-2-yl)-2,5-diphenyltetrazolium Bromide (MTT) Test of Cytotoxicity

Cell survival was determined using the MTT test according to the method of Mosmann, which is modified by Ohno and Abe [43,44]. The MTT test is based on 3-(4,5-dimethylthiazol-2-yl)-2,5-diphenyltetrazolium bromide (MTT) to assess the activity of living cells by their mitochondrial dehydrogenase activity. The nutrient medium used in the experiments was RPM1 1640 medium supplemented with 10% heat-inactivated bovine serum, penicillin (100 IU·mL^−1^), streptomycin (100 µg·mL^−1^), l-glutamine (3 mM) and 25 mM Hepes. In order to analyze the biological effects of HAP/Lig and Ag/HAP/Lig coatings on human cells, cytotoxicity experiments were conducted on human peripheral blood mononuclear cells (PBMC), before and after stimulation to proliferation with mitogen phytohemagglutinin (PHA). The survival of PBMC and PHA-stimulated PBMC in control sample and in the presence of HAP/Lig (1 *wt* % Lig) and Ag/HAP/Lig coatings (1 *wt* % Lig) were investigated 72 h after seeding.

Cell survival (*S*, %) is defined as the ratio of the number of cells grown in nutrient medium with coating and the number of cells grown in control wells containing nutrient medium without coating, multiplied by 100. As the number of live cells is directly proportional to the absorbance of live metabolically active MTT-treated cells, for the calculation of cell survival, absorbance of the newly formed formazan was used instead of the number of live cells (Equation (2)):

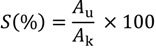
(2)
where *A*_u_ is the absorbance of the cells grown in the presence of a coating and *A*_k_ is the absorbance of the cells of the control sample. The absorbance of the blank was always subtracted from the absorbance of the corresponding cell sample. Experiments were performed in triplicate. The results are reported as the average value ± standard deviation (SD) from three independent experiments.

#### 2.2.10. Antimicrobial Activity

Post-surgical infections of the implantation site are the major problem and inevitably lead to revision surgeries. Antibacterial activities of the samples were tested for microorganisms that are responsible for most of the inter-hospital infections. *Staphylococcus aureus* (Gram-positive bacterium) and *Escherichia coli* (Gram-negative bacterium) can cause serious infections.

The antimicrobial activity of HAP and HAP/Lig (1 *wt* % Lig) coatings on titanium was tested on two bacteria types, bacterial strains *Escherichia coli* (ATCC-25922) and *Staphylococcus aureus* TL (from the culture collection—Faculty of Technology and Metallurgy, University of Belgrade, Serbia) by using agar diffusion method. The experiments were performed in four Petri dishes with nutrient agar (2% agar) cooled to a solid state and used as a base with 4 mm thickness. On the top of thus prepared plates, nutrient soft agar (0.7% agar) was applied containing an indicator strain of a microorganism used for testing antimicrobial activity of coatings. In four small flasks, each containing 15 mL of nutrient agar 200 μL of targeted overnight bacterial culture was added. After agar solidified, HAP and HAP/Lig samples were directly placed. Finally, the agar Petri dishes with samples were incubated for 24 h at 37 °C. The results of antimicrobial activity were estimated by measuring the inhibition zone of bacterial growth formed around the samples (mm).

The antibacterial activity of electrodeposited Ag/HAP/Lig (1 *wt* % Lig) coatings was tested against pathogenic Gram-positive bacteria strain *S. aureus* TL. The culture was maintained through sub-culturing every two months on solid Nutrient medium (Biomedics, Parque Technologico de Madrid, Madrid, Spain) at 37 °C and kept at 4 °C between experiments. Lignin concentration in Ag/HAP/Lig samples was kept at 1 *wt* %. Test bacteria was activated by two successive precultures in LB broth (composition yeast extract 5 g·L^−1^, tryptone 10 g·L^−1^, NaCl 5 g·L^−1^) and incubated at 37 °C during the night. Before the antibacterial test, an overnight culture, not older than 18 h, was diluted in physiological solution (10^−1^) and 2% (*v*/*v*) of culture was used to inoculate a test tube with 7 mL of sterile modified phosphate-buffered (PB) solution (pH = 7.4) with 15 mg titanium coated samples. The initial number of bacteria in each suspension was between 10^4^ and 10^5^ CFU·mL^−1^ and concentration of coating material was approximately 2 mg·mL^−1^. Thus, prepared samples were incubated for 24 h at 37 °C, without shaking. Blank with no titanium coated samples (bacteria in PB solution) was used as control. The number of bacteria in a samples was monitored at the begining of the experiment and after 1 and 24 h of incubation. The samples were serial diluted and 100 µL of appropriate dilutions were mixed with 20 mL of melted LB agar (temperature 55 °C) and poured into Petri dishes. After 24 h incubation at 37 °C, the number of colonies on agar plates containing 25 to 250 colonies were enumerated using a colony counter and expressed as CFU·mL^−1^ to obtain the number of viable *S. aureus*.

## 3. Effect of Lignin Concentration on Non-Sintered and Sintered Hydroxyapatite/Lignin Coatings

The influence of the lignin concentration in the range of 0.5–10 *wt* % Lig on the microstructure, morphology, phase composition, thermal behavior, antimicrobial activity and cytotoxicity of composite HAP/Lig coatings electrodeposited on titanium was investigated in order to find the optimal lignin concentration for producing HAP/Lig composite coatings [32,38].

### 3.1. Surface Morphology and Structural Analysis

TEM image of initial HAP powder (data not shown here) showed that agglomerates are consistent of rod-shaped nanoparticles in the range of 50–100 nm, with Ca/P ratio of 1.67 [42].

**Figure 1:**
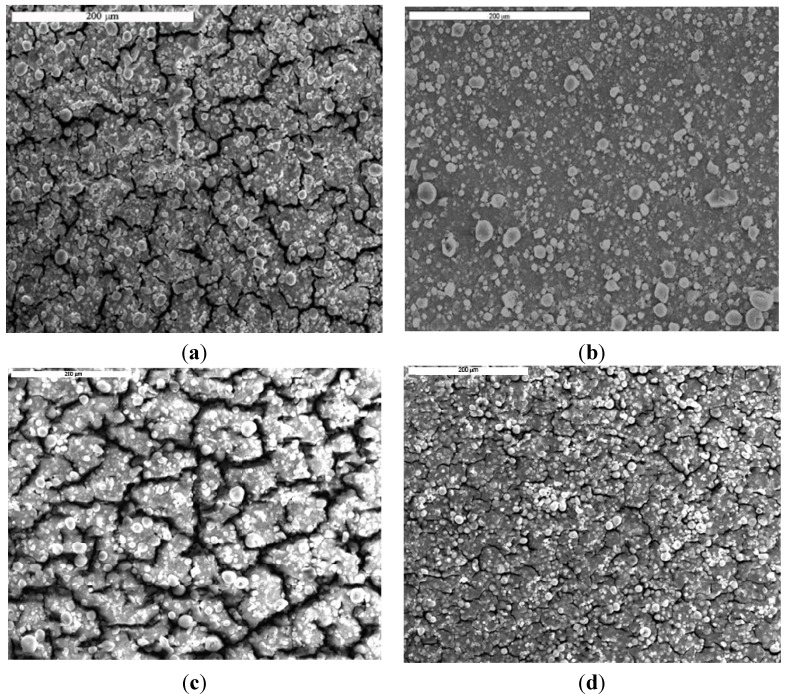
Scanning Electron Microscopy (SEM) micrographs of sintered hydroxyapatite/lignin (HAP/Lig) coatings with: (**a**) 0.5; (**b**) 1; (**c**) 3 and (**d**) 10 *wt* % Lig. Reprinted from [32] with permission. Copyright De Gruyter 2009; and [38] with permission. Copyright Elsevier 2012.

SEM was used to investigate coating surface morphology. SEM micrographs of sintered HAP/Lig (0.5–10) *wt* % Lig coatings deposited under same EPD conditions are shown in Figure 1. Surface of sintered HAP/Lig coatings with 0.5, 3 and 10 *wt* % Lig (Figure 1a,c,d) exhibited large number of cracks on coating surfaces, which may be due to mechanical (HAP–Lig interactions) and thermal stress during sintering [38]. However, SEM micrograph of sintered HAP/Lig coating with 1 *wt* % Lig (Figure 1b) shows homogeneous fracture-free surface. Based on these results the optimal lignin concentration to obtain coatings with a smooth surfaces without fractures was 1 *wt* %. Comparing SEM micrographs of the sintered HAP coatings with different amount of incorporated polymer, HAP/Lig (1 *wt* % Lig) coating stands out indicating that lignin strengthens the bonding between HAP particles and the substrate surface [32].

The possible interactive bonding between HAP and lignin biopolymer was proposed [32]. There are four types of hydrogen bonding that could be proposed. The first is between phenolic hydroxyl –OH from lignin and –OH from HAP; the second is between α-carbonyl from lignin side chain –C=O and –OH from HAP; the third is between –OH from lignin and PO_4_^3−^ from HAP; the last one is between an ether bond oxygen from lignin C–O–C and –OH from HAP.

The phase composition and structure of sintered HAP and HAP/Lig (0.5–10) *wt* % Lig coatings were investigated by XRD analysis. The XRD patterns of the HAP powder used, a pure HAP coating and a HAP/Lig (0.5–10) *wt* % Lig coating, were previously reported [32,38]. All the peaks in XRD patterns of the non-sintered HAP/Lig coatings (0.5–10) *wt* % Lig (data not shown) correspond to the JCPDS pattern No. 09–0432 for hydroxyapatite, confirming the identity of pure hydroxyapatite in the studied coatings. After sintering, the diffraction peaks of HAP/Lig coatings (0.5–10) *wt* % Lig become sharper and of higher intensity with a decrease in peak width, which all indicate that the sintered coatings had a better crystallinity [38]. A higher degree of crystallinity would make coatings less prone to dissolution in body fluids [45]. The sintering depends on the characteristics of the initial HAP powder, and the smaller particles have a tendency to aggregate in order to minimize their high free surface energy, resulting in densification and an increase in the grain size [46,47]. Therefore, the initial nanosized HAP powder can be successfully sintered at temperature of 900 °C and that particular chosen temperature was applied as thermal treatment of the composite HAP/Lig coatings, although the usually applied sintering temperature is in the range between 1000 and 1300 °C [48].

**Figure 2:**
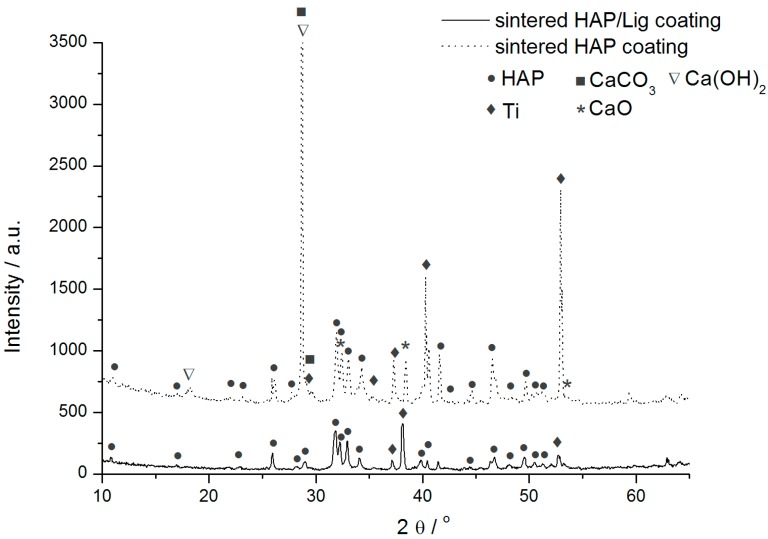
X-ray diffraction (XRD) patterns of sintered HAP and HAP/Lig (1 *wt* % Lig) coatings.

By comparing the XRD patterns of sintered HAP and HAP/Lig coatings (0.5–10 *wt* % Lig), the partial HAP decomposition during thermal treatment at 900 °C was confirmed for HAP and HAP/Lig with 0.5 *wt* % Lig (data not shown) [32,38]. The differences between XRD patterns of HAP and HAP/Lig coating (1 *wt* % Lig) are presented in Figure 2. While the main crystalline phase of pure sintered HAP coatings was still HAP, the observed new diffraction peaks indicated the formation of crystalline phases of CaO and CaCO_3_ (Figure 2). It can be explained that the reaction of CaO, generated during HAP sintering, with traces of atmospheric water and CO_2_, yields Ca(OH)_2_ and carbonate, respectively [49]. In the case of sintered HAP/Lig coating with 0.5 *wt* % Lig, beside these peaks a new phase TiP appeared [38]. The presence of a specific TiP peak could be explained by the diffusion of phosphorous ions into the Ti surface as a result of HAP decomposition. It was reported that during the thermal process, the diffusion of calcium (limited diffusion) and phosphorus (profuse diffusion) ions into the Ti substrate can occur, resulting in the HAP block decomposition [38,50]. But, on the other hand, the specific diffraction peak for β-Ca_3_(PO_4_)_2_, (TCP, tricalcium phosphate) at 2θ = 30.7° was absent in all XRD spectra [38,51].

On the contrary, diffractograms of the sintered HAP/Lig coatings containing 1, 3 and 10 *wt* % Lig indicated only HAP (data not shown). Comparing the diffraction patterns of all sintered HAP/Lig coatings, it can be concluded that degradation of hydroxyapatite did not occur for lignin concentrations of 1 *wt* % and higher, meaning that higher concentrations of lignin protect HAP lattice from decomposition during sintering. The mean crystallite domain size (*D*_p_) of the HAP/Lig coatings (0.5–10) *wt* % Lig was calculated using the Scherrer Equation (1).

The values of mean crystallite domain size were calculated to be between 35 and 39 nm for HAP/Lig coatings with 0.5, 1, 3 and 10 *wt* % Lig, indicating that mean crystallite domain size does not depend on the lignin concentration.

### 3.2. XPS and ATR-FTIR Analyses

The qualitative analysis of the XPS spectra of studied HAP and HAP/Lig (0.5–10) *wt* % Lig coatings revealed certain differences between non-sintered and sintered coatings. From spectra Ca 2p, P 2p, O 1s, and C 1s peaks were observed whereas an additional peak related to Ti 2p appeared for the sintered HAP coating and could be assigned to: (1) TiO_2_ (binding energy ~459 eV); (2) surface fracture; and/or (3) phosphorus ions diffusion into Ti surface. We could also observe trace amounts of other elements in the studied samples. The quantitative analysis data of the XPS spectra obtained from high-resolution measurements for sintered HAP and HAP/Lig with lignin concentrations of 0.5, 1, 3 and 10 *wt* % Lig are presented in Table 1 [32,38].

**Table 1:** Quantitative XPS analysis data for HAP and HAP/Lig (0.5–10) *wt* % Lig coatings Reprinted from [38] with permission. Copyright Elsevier 2012.

HAP/Lig, *wt* % Lig	Thermal Treatment	Ca	P	C	Ca/P
HAP	non-sintered	19.4	11.3	7.2	1.72
	sintered	16.5	5.5	21.7	3.00
0.5	non-sintered	19.1	11.3	8.2	1.69
	sintered	18.4	7.9	15.9	2.33
1	non-sintered	19.3	10.8	10.5	1.79
	sintered	18.7	8.9	11.3	2.10
3	non-sintered	18.4	12.0	11.7	1.53
	sintered	18.8	10.8	12.9	1.74
10	non-sintered	15,8	10,3	21,3	1.53
	sintered	17,1	9,6	18,9	1.78

All the samples contain C 1s, even pure HAP coating, which is supposed to be free of organic material. This is due to the common organic contamination from the environment (adventitious carbon) on the substrate surface. It is well known that an impurity is often incorporated into synthetic apatite due to the presence of CO_2_ in the air or in solution. However, the carbon content of non-sintered HAP/Lig (1 *wt* % Lig) coatings is higher than that of non-sintered HAP coating, which confirms the presence of lignin. On the other hand, the carbon content of the non-sintered HAP/Lig (0.5–10) *wt* % Lig coatings increased with increasing Lig concentration, which confirmed that lignin was bound to the HAP lattice [38].

**Figure 3:**
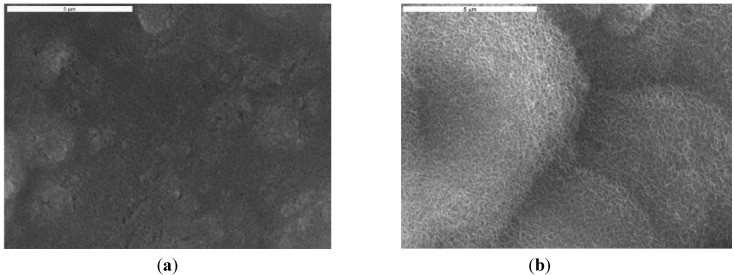
SEM micrographs of sintered Ag/HAP/Lig coating (**a**) before and (**b**) after immersion in SBF solution at 37 °C. Scale bar: 5 µm. Reprinted from [39] with permission. Copyright American Chemical Society 2013.

However, the highest increase in the carbon content after sintering was evidenced for pure HAP and HAP/Lig coating with 0.5 *wt* % Lig, which could be explained by carbon from CaCO_3_ formed by reaction between CaO with atmospheric CO_2_. The HAP decomposition and the presence of CaO and CaCO_3_ after sintering were also confirmed in the XRD patterns of the same coatings (Figure 2). A small increase in the carbon content of the sintered HAP/Lig coatings with 1 and 3 *wt* % Lig compared to non-sintered coatings indicates the absence of HAP decomposition during sintering, which confirms that lignin limits the formation of CaO. The decrease in carbon content after sintering for the HAP/Lig coating with 10 *wt* % Lig indicates the highest weight loss of lignin. The absence of new diffraction peaks for CaO and Ca(OH)_2_ in XRD patterns of sintered HAP/Lig coatings with 1 (Figure 2), 3 and 10 *wt* % Lig (data not shown here) also indicates that there is no degradation of hydroxyapatite lattice.

The calculated Ca/P ratio varied in the range of 1.53–1.69 for the non-sintered HAP and HAP/Lig coatings, which is similar to the Ca/P ratio for stoichiometric HAP (1.67). According to the literature [52], stable HAP phases correspond to Ca/P ratio within a range of 1.3–1.8. It could be observed that the Ca/P ratios of the sintered HAP/Lig coatings were higher than those of the non-sintered (Table 1). The highest increase in Ca/P ratio to 3.00 for sintered HAP coating and 2.33 for sintered HAP/Lig coating with 0.5 *wt* % Lig can be explained by diffusion of phosphorus ions, resulting in the partial HAP decomposition and also the presence of a new TiP peak in XRD pattern (data not shown here). It could be concluded that lignin limited the decomposition of the HAP lattice of sintered HAP/Lig coatings with (1–10) *wt* % Lig as indicated by the smaller increase in carbon content and smaller Ca/P ratio, compared to pure HAP coating and HAP/Lig coating with 0.5 *wt* % Lig. This was also confirmed by XRD results.

ATR-FTIR measurements (data not shown here) were used to identify and verify the presence of specific functional groups on the surfaces of non-sintered and sintered HAP and HAP/Lig (0.5–10) *wt* % Lig coatings [32,38]. The spectra of non-sintered HAP and HAP/Lig coatings with different lignin concentration, exhibit characteristic ν_1_PO_4_^3−^, ν_3_PO_4_^3−^ and ν_4_PO_4_^3−^ bands typical for the PO_4_^3−^ group. In addition, the characteristic band at 630 cm^−1^ corresponds to the vibration of structural OH^−^ groups [23]. The entire ATR-FTIR spectra indicate the absence of carbonate bands in the range of 1500–1400 cm^−1^ [53], confirming that hydroxyapatite powder is pure HAP phase.

The ATR-FTIR spectra of HAP/Lig coatings before sintering are very similar to the ATR-FTIR spectrum of pure HAP coating with respect to the functional groups, which indicates that the lignin in HAP/Lig coatings does not significantly alter the structure of the hydroxyapatite lattice [32,38]. However, in the ATR-FTIR spectrum of HAP/Lig coatings, the appearance of C–H deformation vibration corresponds to C–H bonds in the aromatic rings [38,54], as well as to bands for the methoxy group of lignin [38,55]. This implies that lignin presence in the HAP/Lig coating does not change the formation and structure of the HAP lattice [32,38].

Most of the lignin hydroxyl groups are phenolic hydroxyl groups, which have a strong ability to form hydrogen bonds with the carbonyl groups; this formation of hydrogen bonds would induce an obvious shift of the band to lower wavenumbers. This can be considered as evidence for the formation of intermolecular hydrogen bonds. The appearance of ν(O–H) vibrations in OH^−^ groups from HAP, occurred at a lower wavelength than expected (data not shown), confirming intermolecular hydrogen bonding between hydroxyapatite and lignin [38,53]. According to the literature, it could be proposed that inter hydrogen bonds (P–O···OH) between OH^−^ groups from lignin and PO_4_^3−^ groups from hydroxyapatite were established as evidenced by phosphate bands appearing at lower wavenumber [38]. These two observations proved our previously proposed model of hydrogen bonding between hydroxyapatite and lignin [32].

Comparing the ATR-FTIR spectra for sintered HAP/Lig (0.5–10) *wt* % Lig coatings (data not shown here), it was observed that HAP decomposition during sintering does not occur for coatings with lignin concentration 1, 3 and 10 *wt* % Lig [32,38]. In other words, lignin concentrations of 1 *wt* % and higher prevent HAP decomposition and/or diffusion of phosphorus ions into the Ti surface due to the established hydrogen bonds.

It can be concluded that lignin concentrations of 1 *wt* % and higher protect hydroxyapatite lattice during sintering, which was confirmed by XRD, XPS and ATR-FTIR results. Therefore, based on the above-mentioned result and SEM micrographs, the optimal lignin concentration used for mechanical and biological testing was 1 *wt* % Lig in order to obtain non-toxic and antimicrobial biomaterial for bone tissue engineering.

### 3.3. Nanoindentation Test

Nano-indentation technique has became a method of choice for studies of bicomposite coating materials due to its many advantages: test depth can be less than coating thickness; the nanomechanical response of the coated system can be measured on the substrate-*in situ*; and tests can be performed at variable displacements. The technique thus represents a unique way of ascertaining elastic, plastic and fracture responses of surfaces [56].

The elastic modulus and hardness of sintered HAP and HAP/Lig (1 *wt* % Lig) coatings were analyzed by Nanoindentation test. The mean hardness, *H*, was measured to be 7.40 GPa for the HAP coating, while the mean reduced elastic modulus, *E*_r_, was found to be 132 GPa. These values are in good agreement with published data [57] for bulk hydroxyapatite (*H* = 6.19–6.76 GPa and *E*_r_ = 122–125 GPa), as well as for thin film coatings. The HAP/Lig (1 *wt* % Lig) coating showed mean hardness of 6.90 GPa and mean reduced elastic modulus of 134 GPa, which were comparable to those of the pure HAP coating.

In general, for biomedical metallic implants, such as total hip and knee replacements, the bonding strength (or inter-laminar shear strength) between implant and coating layer is the most important issue for lifespan of replaced implant [58]. Therefore, the obtained *E*_r_ and *H* results for HAP/Lig biocomposite leads us to the conclusion that lignin does not significantly affect the mechanical properties of the composite, probably due to small concentrations of incorporated lignin.

### 3.4. Biological Tests

#### 3.4.1. Cytotoxicity—MTT Test

Cellular response of the host organism, estimated by their viability in the presence of implant materials, were tested by the standardized MTT test to assess the activity of living cells by their mitochondrial dehydrogenase activity.

The survival of PBMC stimulated to proliferate with mitogen phytohemagglutinin (PHA) in the control sample and in the presence of sintered HAP/Lig coatings (1 *wt* % Lig) were investigated 72 h after seeding. The results presented in Table 2 show survival rate of the PBMC and PHA-stimulated PBMC in the presence of HAP/Lig coating with 1 *wt* % Lig [38].

MTT results indicate that HAP/Lig coating could induce a mild decrease in survival of healthy immunocompetent PHA-stimulated PBMC, but that the results were similar to that of the control sample (*S* = 100%). According to the literature [59], HAP/Lig coating can be classified as non-toxic for PHA-stimulated PBMC, while as slightly cytotoxic for unstimulated PBMC.

The reason for the slight lignin cytotoxicity could be due to its known absorption capability, *i.e*., it could slightly non-specifically absorb some of the micronutrient constituents needed for sustaining tested PBMC proliferation, but also could be due to lignin antioxidant activity, as shown in tests on human keratinocytes and mouse fibroblasts [60]. On the other hand, one can speculate that lignin structures became more condensed upon sintering, which could have led to formation of highly condensed aromatic structures, known for their toxicity.

**Table 2:** Cell survival of peripheral blood mononuclear cells (PBMC) and PBMC phytohemagglutinin (PHA)-stimulated in the presence of sintered HAP/Lig (1 *wt* % Lig) coatings Reprinted from [38] with permission. Copyright Elsevier 2012.

**Cell Type**	**Peripheral Blood Mononuclear Cells (PBMC)**
Material	HAP/Lig coating, 1 *wt* % Lig
Cell viability (*S*), %	65.9 ± 18.3
Classification	Slightly cytotoxic
	**PHA-Stimulated Peripheral Blood Mononuclear Cells (PBMC + PHA)**
Material	HAP/Lig coating, 1 *wt* % Lig
Cell viability (*S*), %	90.4 ± 8.2
Classification	Non-cytotoxic

#### 3.4.2. Antimicrobial Activity

The antimicrobial activity of HAP and HAP/Lig (1 *wt* % Lig) coatings on titanium was tested on bacterial strains *E. coli* and *S. aureus* by using the agar diffusion method. There was no light zone around HAP and HAP/Lig coatings (1 *wt* % Lig) after 24 h of incubation in the case of both *S. aureus* and *E. coli*. After removing titanium coated samples from the agar surface, inhibited zones of bacteria growth were observed for bacterial cultures of both *S. aureus* and *E. coli*, but only below the sample, not around it. Based on the lack of zones of bacterial growth inhibition, the conclusion was that HAP and HAP/Lig coatings do not possess antimicrobial activity necessary for the protection of the human body.

These results indicate that the presence of lignin has no significant effect on the antimicrobial properties of hydroxyapatite. Antibacterial effect of HAP/Lig coating is similar to the effect of the HAP coating since in both cases the zone of inhibition of bacterial growth around the surveyed coating were missing. Since it was observed that under the coating zone inhibited growth of bacteria exists, it can be assumed that the diffusion through the agar surface was accelerated by gravitational force.

However, the main problem of HAP/Lig coatings with 1 *wt* % Lig was the lack of antimicrobial activity that was necessary for implant protection during the initial period of implantation. Therefore, the subsequent course of research aimed at obtaining biocomposite coatings doped with silver ions, a major antimicrobial agent. The ultimate goal was to obtain a compact, well-fitting composite Ag/HAP/Lig coatings with 1 *wt* % Lig with excellent antimicrobial properties, corrosion stability in SBF solution and non-toxic to human cells.

## 4. Electrophoretically Deposited Silver/Hydroxyapatite/Lignin Coatings

### 4.1. Bioactivity of Silver/Hydroxyapatite/Lignin Coatings

The influence of silver on the microstructure, morphology, phase composition, thermal behavior, antimicrobial activity and cytotoxicity, as well as on corrosion stability and bioactivity of silver/hydroxyapatite/lignin (Ag/HAP/Lig) coating with 1 *wt* % Lig electrodeposited on titanium in SBF, was investigated [40].

### 4.2. Surface and Structural Analysis of Ag/HAP/Lig Coating before and after Immersion in Simulated Body Fluid (SBF) Solution

Surface morphology of the sintered Ag/HAP/Lig coating before and after 7 days of immersion in SBF at 37 °C is shown in Figure 3. The SEM micrograph (Figure 3a) revealed smooth and uniform surface of Ag/HAP/Lig coating with no fractures before soaking in SBF, while Figure 3b represents coating surface after immersion in SBF. Newly formed plate-shaped apatite crystals are evident in Figure 3b after immersion in SBF solution. A relatively high porosity of implant surface along with improved mechanical stability provides better cell adhesion that facilitates osteointegration. The key point is that high interconnected porosity structures enable the penetration of osteoblasts leading to better connection between the implant and the bone [10].

Obvious potential bioactivity was confirmed by forming an apatite layer on the coating surface after 7 days of immersion in SBF. The composition of new plate-shaped crystals was revealed in ATR-FTIR and XRD spectrum analysis (stated below) [39], while the formation of apatite has been previously explained by Sun *et al.* [61]. The negatively charged hydroxyapatite surface interacts with Ca^2+^ ions from SBF forming an amorphous positive Ca-rich surface. Subsequently, thus formed surfaces interact with the negative PO_4_^3−^ ions in the SBF to form Ca-poor apatite, which gradually crystallizes into bone-like apatite. Once formed in SBF, the apatite grows spontaneously, consuming the calcium and phosphate ions, incorporating minor ions, such as sodium, magnesium, and carbonate, and thereby developing a bone mineral-like compositional and structural feature [62].

**Figure 4:**
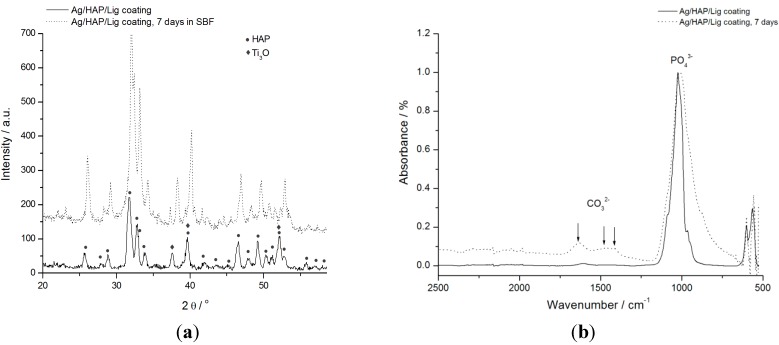
(**a**) XRD patterns and (**b**) attenuated total reflection Fourier transform infrared spectroscopy (ATR-FTIR) spectra of sintered Ag/HAP/Lig coating, before and after 7 days immersion in SBF at 37 °C. Reprinted from [39] with permission. Copyright American Chemical Society 2013.

XRD analysis was performed to determine the phase composition and structure of Ag/HAP/Lig coatings before and after immersion in SBF, presented in Figure 4a. XRD diffractogram of Ag/HAP/Lig coating before immersion in SBF was very clean showing only characteristic hydroxyapatite peaks without any additional crystalline phases even after sintering, meaning that lignin protected also Ag/HAP lattice during sintering [39].

Labeled XRD peaks match very well the JCPDS standard XRD card No. 86-1199 for hydroxyapatite, but incorporation of Ag in the hydroxyapatite crystal lattice causes a shift of specific HAP peaks toward smaller 2θ values, confirming the silver substitution for calcium [39]. The evidence of Ag presence was verified through the shift of characteristic HAP peaks (crystal planes (002), (211), (112), and (300)) toward smaller angles for the Ag/HAP coating before immersion in SBF solution compared to the pure HAP coating (Figure 4a). The additional peaks that originate from the Ti substrate indicate the presence of the suboxide of titanium, Ti_3_O (JCPDS No. 73-1586), as seen in Figure 4a, which is classified as a nonstoichiometric oxide deficient in oxygen. The complex valence status of Ti appears to be due to the oxygen diffusion from the exterior surface to the inside during sintering. According to Ye *et al.* [63] Ti metal and Ti suboxides on the composite surfaces are believed to be more active than TiO_2_ in the physiological environment and can activate chemical bonding between the implant surface and adjacent biomolecules.

After 7 days of immersion in SBF solution, a new phase was detected by observing the shift in characteristic hydroxyapatite peaks toward higher angles (Figure 4a). These findings were attributed to carbonate ions in the lattice and confirmed, therefore, the growth of carbonate hydroxyapatite onto the surface of Ag/HAP/Lig coating. Therefore, the shifting of XRD diffraction peaks is typical for weak crystalline, carbonated HAP, as it is found in bone.

The mean crystallite domain size (*D*_p_) was calculated at 2θ ≈ 26° by the Scherrer Equation (1). The crystallite domain size of Ag/HAP/Lig coatings before and after soaking in SBF solution was calculated to be almost the same, 20.8 and 22.0 nm, respectively, indicating the homogeneous surface. Small difference in value between the crystallite size before and after immersion is probably due to the incorporation of CO_3_^2−^ ions into the apatite lattice by occupying the OH^−^ sites or the PO_4_^3−^ positions [64].

The bioactivity of implanted apatite coated materials can be evaluated by the formation of bone-like apatite on their surface. The presence of CO_3_^2−^ bands in ATR-FTIR spectrum is clear evidence of its incorporation in the HAP layer. It is well known that the biological hydroxyapatite also contains carbonate groups [65]. Thus, Ag/HAP/Lig coating was investigated by the ATR-FTIR method before and after immersion in SBF solution as shown in Figure 4b.

As it can be seen before immersion in SBF, the ATR-FTIR spectrum exhibited characteristic hydroxyapatite bands as found in the literature [66,67]; namely, the presence of phosphate groups was confirmed by vibrational bands at 960, 1016 and 1089 cm^−1^ in the FTIR spectrum. Also, the weak characteristic bands at around 3573 and 627 cm^−1^ correspond to the vibration of structural OH– groups found in the hydroxyapatite lattice. The absence of a low-intensity wide band at wavenumbers between 1400 and 1585 cm^−1^ in the ATR-FTIR spectrum of Ag/HAP/Lig before immersion in SBF solution (Figure 4b) confirmed that there was no decomposition of hydroxyapatite, as also detected by XRD diffractogram (Figure 4a).

ATR-FTIR analysis, as an efficient and sensitive method for detection of small amounts of carbonates [68], was used to investigate Ag/HAP/Lig coating surface after 7 days of soaking in SBF solution. During 7 days of immersion in SBF at 37 °C, carbonated apatite was formed on the surface of coatings, as revealed by ATR-FTIR spectrum (Figure 4b). This spectrum revealed the broad absorbance band at 3382 cm^−1^ attributed to the OH^−^ group stretching with higher intensity than the intensity before immersion, revealing the formation of new bone-like apatite layer on the coating surface [61]. Also, a further confirmation was made through observation of three peaks at 1640, 1476 and 1420 cm^−1^, attributed to the vibrational bands of CO_3_^2−^ groups. According to the literature, B-type carbonated apatite appears on the surface after soaking in SBF solution [63,69,70]. The spectrum of bone-like apatite showed a high concentration of OH^−^ and PO_4_^3−^ groups compared to the peaks appearing in the spectrum of Ag/HAP/Lig coating before immersion in SBF, which allows the coating surface to exhibit the negative surface potentials required for apatite nucleation.

All of the above results: SEM, ATR-FTIR and XRD (Figure 3 and Figure 4), proved the bioactive nature of Ag/HAP/Lig coating surface by the growth of the carbonate hydroxyapatite layer on its top after 7 days of immersion in SBF at 37 °C. Therefore, the formation of carbonated HAP is very beneficial due to its weak crystalline form that resembles human bone, a property that facilities osteointegration [68].

The chemical composition of the outermost coating surface level is important because it would be in direct contact with the bone tissue and dissolve first at the initial stage of implantation. It has been shown that the optimum Ca/P ratio is 1.67–1.76 [71]. From semi-quantitative XPS analysis of Ag/HAP/Lig coating, Ca/P ratio was calculated to be 1.62 [39], which is close in value to the Ca/P ratio in stoichiometric hydroxyapatite (1.67). Thus, Ca/P ratio for the Ag/HAP/Lig coating after sintering remains constant (1.62) confirming the lignin protection of Ag/HAP lattice during sintering [71].

### 4.3. Corrosion Stability of Ag/HAP/Lig Coatings in SBF

EIS measurements were employed in investigation of corrosion stability of Ag/HAP/Lig coating in the physiological environment such as the SBF solution. The Nyquist plots for the impedance of Ag/HAP/Lig coating deposited on titanium after a prolonged exposure time in SBF solution at 37 °C were obtained, where the high-frequency range is attributed to the coating, while the low frequency range describes the characteristics of the passive oxide layer on titanium [39].

**Figure 5:**
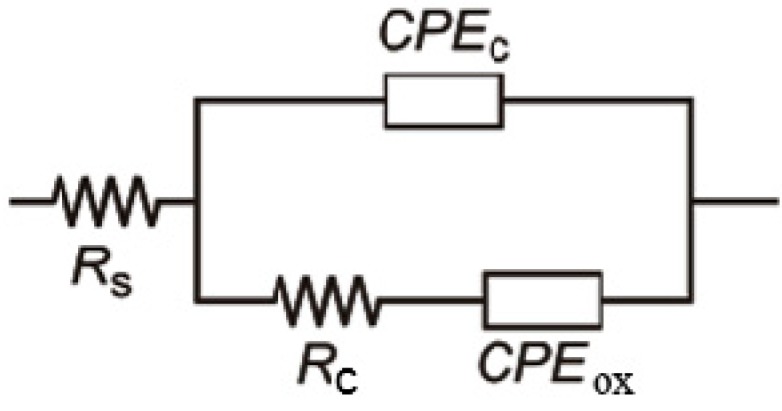
Equivalent electrical circuit for sintered Ag/HAP/Lig coating on titanium during initial and prolonged time of SBF exposure at 37 °C. Reprinted from [39] with permission. Copyright American Chemical Society 2013.

Fitting of experimental data obtained from Nyquist plots for 14 days in SBF was accomplished by using the equivalent electrical circuits (EEC) shown in Figure 5, using Gamry Instruments Echem Analyst fitting program. EEC consists of the electrolyte resistance, *R*_s_, the coating pore resistance, *R*_c_, and constant phase elements, CPE_c_ and CPE_ox_, which represent all the frequency-dependent electrochemical phenomena, such as the coating capacitance, *C*_c_, and passive oxide film capacitance, *C*_ox_. CPE is used in these models to compensate non-homogeneity in the system and is defined by two parameters, *Y*_0_ and *n*. The impedance of CPE is represented by the following Equation (3) [72,73,74]:

*Z*_CPE_ = *Y*_0_^−1^ × (*j*ω)^−*n*^ (3)

where *j* = (−1)^1/2^, ω = 2π*f* is frequency in rad·s^−1^ and *f* is the frequency in Hz. If *n* values range from 0.8 to 1, the impedance of CPE can be considered to be the one of the pure capacitor (Equation (4)):

*Z*_CPE_ = (*j*ω*C*)^−*n*^ (4)

In this case *Y*_0_ gives a pure capacitance (*C*). The impedance data in the complex plane were well fitted by the proposed EEC and we used three basic criteria to evaluate the general accuracy of the fit: visual fit to Nyquist plots, low goodness of fit and low relative standard errors for every circuit element [75]. We obtained suitably low goodness of fit, GOF (<10^−4^) and the error associated with each element was lower than 10%. It can be concluded that the chosen fit describes investigated systems accurately. The obtained fitting results are listed in Table 3.

**Table 3:** The fitting values of equivalent electrical circuits for sintered Ag/HAP/Lig coating. Reprinted from [39] with permission. Copyright American Chemical Society 2013.

Sample	*t*/h	*R*_s_/Ω cm^2^	CPE_ox_ (*C*_ox_)/μF·cm^−2^	*n*_ox_	CPE_c_ (*C*_c_)*/*μF·cm^−2^	*n*_c_	*R*_c_/kΩ cm^2^
**Ag/HAP/Lig**	1	43.3	1030.0	0.76	745.2	0.88	4.3
	3	44.5	1046.0	0.80	697.6	0.88	5.2
	6	44.5	1010.0	0.81	667.9	0.88	5.9
	8	44.1	880.1	0.77	655.8	0.88	6.1
	24	29.2	620.8	0.70	627.2	0.88	5.6
	72	23.1	821.3	0.76	588.2	0.88	6.4
	120	31.5	610.4	0.74	560.6	0.89	5.9
	168	18.8	782.4	0.77	559.3	0.88	5.8
	240	21.8	522.0	0.74	543.5	0.88	6.3
	288	21.3	475.8	0.70	529.0	0.88	6.9
	336	17.7	403.2	0.71	547.1	0.87	6.3

As seen in Table 3, *n*_c_ and *n*_ox_ values for Ag/HAP/Lig coating are close to 0.8, therefore CPE_c_ can be considered as coating capacitance, *C*_c_, while CPE_ox_ can be considered as capacitance of oxide film on the titanium surface beneath Ag/HAP/Lig coating, *C*_ox_. The time dependence of coating pore resistance, *R*_c_, and the coating capacitance, *C*_c_, of the Ag/HAP/Lig coating during 14 days in SBF are presented in Table 3. It can be noticed that the pore resistance, *R*_c_, increased and coating capacitance, *C*_c_, decreased during the exposure to SBF, which is related to the growth of the newly formed apatite layer on the coating surface. The continuous increase in *R*_c_ up to 6.3 kΩ cm^2^ and decrease in *C*_c_ up to 547.1 μF·cm^−2^ reflect the process of the apatite nucleation after prolonged time in SBF, which was clearly seen in SEM images (Figure 3), as well as from XRD and ATR-FTIR (Figure 4). This suggests that Ag/HAP/Lig coating surface represented the site of nucleation and growth of new apatite layer. According to the literature, transformation of hydroxyapatite with a bone-like crystallinity apatite layer in the human body certainly induces stable bonding to bone [76].

### 4.4. Nanoindentation Test

Ag/HAP coating had reduced elastic modulus, *E*_r_, and mean hardness, *H*, of 172 and 14.5 GPa, respectively, whilst Ag/HAP coating with 1 *wt* % lignin had slightly higher *H* and lower *E*_r_ values of 173 and 13.3 GPa, respectively. Comparing HAP and HAP/Lig coating and their counterparts with silver, it was observed that addition of Ag contributed to the increase of both *E*_r_ (172 *vs*. 132 GPa) and *H* (14.5 *vs*. 7.40 GPa) of the composites.

An evaluation of *E*/*H* value is a prerequisite for the evaluation of fracture toughness [77]. In the case of pure HAP and Ag/HAP coatings, *E*_r_/*H* ratios were 17.86 and 11.87, respectively, while in the case of HAP/Lig and Ag/HAP/Lig coatings *E*_r_/*H* ratios were 19.34 and 13.00, respectively, indicating that Ag reinforcement caused decrements in the value of *E*_r_/*H*, implying that toughness values may be affected with Ag addition. On the other hand, calculated *E*_r_/*H* ratios for HAP and HAP/Lig coatings were 17.86 *vs.* 19.34, respectively, for Ag/HAP and Ag/HAP/Lig the same comparison in *E*_r_/*H* ratio was noticed 11.87 *vs.* 13.00, respectively. Therefore, we have concluded that the change in *E*_r_/*H* ratio was not significant for pure coatings *vs.* coatings with incorporated lignin. Therefore, small concentration of 1 *wt* % lignin does not affect mechanical properties of composite.

The relationship between material surface properties and biological response to it, is a major issue in biomedical materials research. The Ag/HAP/Lig composite can serve as a potential biomaterial bacause Ag addition improves its bactericidal property, since it was shown that HAP/Lig does not prevent bacterial growth. Although incorporated silver affects mechanical properties, its main role is the improvement of antibacterial properties. To evaluate bactericidal effect and toxicity toward human cells, bacteria culture tests and citotoxicity evaluations were conducted and results are discussed below.

### 4.5. Biological Tests

#### 4.5.1. Silver Release

Silver release into the physiological environment plays an important role in the antibacterial efficiency [78]. The coatings doped with silver ions provided high initial concentration of antimicrobial agent in surrounding tissue. This property is especially important in the early critical post-implantation period since it prevents initial adhesion of bacteria [79]. However, continuous silver ion release after this critical period is also desirable to prevent bacteria biofilm formation. Concentration of silver ions released from the Ag/HAP/Lig coating during 10 days in SBF solution at 37 °C was investigated by atomic absorption spectroscopy (AAS)-inductively coupled plasma (ICP) spectrometry [40]. The cumulative silver ion release from the Ag/HAP/Lig coating after 10 days was measured to be 1.704 ppm. Jamuna-Thevi *et al.* previously reported that the minimum effective silver ion concentration is 0.1 ppb and the maximum cytotoxic concentration toward human cells is 10 ppm [79], therefore our measured concentrations were within this range. The concentration of silver released from Ag/HAP/Lig coating was more than previously published initial antibacterial concentration of silver, found to be 56 ppb [79].

#### 4.5.2. Cytotoxicity—MTT Test

Cytotoxicity of Ag/HAP/Lig (1 *wt* % Lig) coating was determined by MTT test against PBMC and PHA-stimulated PBMC cells (Table 4) [40]. PBMC consist of lymphocytes and monocytes, thus representing one of the main populations of human immune system cells [80]. For our initial testing, we decided to investigate the effects of coatings on PBMC as the first line of immune defense against any type of implant in the human body, before proceeding to tissue specific cells (osteoblasts).

It is very important to develop biomaterials that will not exert toxic effects against cells of the surrounding tissue as well as against healthy immunocompetent PBMC, components of the immune response. Examination of cytotoxic effects of the investigated Ag/HAP/Lig coatings showed mild decrease in survival of healthy immunocompetent PBMC, unstimulated (89.5%) and PHA-stimulated (83.8%) compared to the control cell sample (*S* = 100%). According to the literature [59], Ag/HAP/Lig coating with 1 *wt* % Lig was displayed as non-cytotoxic against target PBMC cells.

**Table 4:** Cell survival of PBMC and PBMC PHA-stimulated in the presence of sintered Ag/HAP/Lig coating.

**Cell Type**	**Peripheral Blood Mononuclear Cells (PBMC)**
Material	Ag/HAP/Lig coating, 1 *wt* % Lig
Cell viability (*S*), %	89.4 ± 3.5
Classification	Non-cytotoxic
**Cell Type**	**PHA-Stimulated Peripheral Blood Mononuclear Cells (PBMC + PHA)**
Material	Ag/HAP/Lig coating, 1 *wt* % Lig
Cell viability (*S*), %	83.8 ± 6.3
Classification	Non-cytotoxic

#### 4.5.3. Antimicrobial Activity

Recently, research in orthopedic surgery has focused on the development of surface modified devices that are capable of releasing drugs adapted to the clinical situation (antibiotics, antimicrobial agents *etc*.) in a controlled and predictable manner, according to established kinetic laws. Silver, including Ag ions and Ag nanoparticles, are well-known as primary inorganic antimicrobial agents that have been widely used in different fields of medicine. Although not completely revealed, it is assumed that Ag ion disrupts the bacterial cell integrity by binding to the enzymes and proteins within the bacteria, thus accelerating their death. In this study [40], the antibacterial effect of Ag/HAP/Lig (1 *wt* % Lig) coatings were investigated against *S. aureus*, a species of bacteria frequently responsible for post-surgical infections in orthopedic surgery [81].

Antibacterial activity was investigated quantitatively by monitoring changes in the viable number of bacterial cells in suspension. Table 5 depicts the antibacterial activity of Ag/HAP/Lig coating against strain *S. aureus* TL in PB solution.

According to Table 5, antimicrobial activity of the Ag/HAP/Lig coating could be noticed immediately after inoculation of samples and further reduction of cell viability for two logarithmic units is achieved after just 1 h of incubation when compared to the initial number of cells in suspensions (percentage of cell reduction was 97.67%). Based on silver ion release results, the concentration of silver ions after 1 h was 0.4493 ppm [40], which is a sufficiently small concentration to achieve antibacterial effect without causing cytotoxicity (Table 4).

**Table 5:** Reduction of viable cell number of *S. aureus* TL after incubation with Ag/HAP/Lig coating for 0, 1 and 24 h.

Bacteria Strain Type	*S. aureus* TL.		
	Initial	1 h	24 h
Control [CFU·mL^−1^]	1.0 × 10^5^	3.0 ×10^4^	9.9 × 10^4^
Ag/HAP/Lig [CFU·mL^−1^]	2.5 × 10^4^	2.0 × 10^3^	No bacteria

In comparison to the reported results of Stanic *et al.* that refer to silver release from hydroxyapatite powders [82], the antimicrobial efficiency of Ag/HAP/Lig coating exhibited higher reduction of bacteria strain *S. aureus* TL, since after 24 h, analyzed samples did not contain any viable cells and visible colonies were not detected in the samples directly taken from suspension. According to the antibacterial assays, the antibacterial efficacy of the sample that contained Ag ion (Ag/HAP/Lig coating) was very similar as can be seen from the kinetics of cell killing presented. The total reduction in the bacterial numbers after 24 h indicates antimicrobial activity of 0.5 *wt* % Ag in Ag/HAP/Lig coating, providing good protection against infection. Based on our results, an immediate silver ion release provides for the imminent drop in CFU numbers even after 1 h of exposure, which is the bactericidal effect needed for prevention of biofilm formation [79].

## 5. Conclusions

Composite HAP/Lig and Ag/HAP/Lig coatings were successfully obtained by electrophoretic deposition (EPD) method using constant voltage method at optimized parameters 60 V for 45 s with sufficient thickness, good adhesion and mechanical properties and uniform surfaces without phase transformation. Novel lignin-based coatings were successfully sintered at a temperature of 900 °C, which is considerably lower than the usual sintering temperature (1000–1300 °C), which is a result of the use of nanosized HAP and Ag/HAP powders. It is proposed that the biopolymer lignin allows the formation of a compact, well-adherent and homogeneous coating due to the establishment of different hydrogen bonds between the functional groups of hydroxyapatite and lignin.

Based on XRD, ATR-FTIR and XPS results, it was confirmed that lignin concentrations of 1 *wt* % and higher protect hydroxyapatite lattice from decomposition during sintering. Quantitative XPS measurements showed that the Ca/P ratio for non-sintered HAP/Lig coatings (1.79) is similar to the stoichiometric Ca/P ratio (1.67). After sintering there is an increase in the Ca/P ratio in the case of sintered HAP and the HAP/Lig coating with 0.5 *wt* % Lig as a result of the diffusion of phosphorus ions in the lattice of HAP to titanium, which confirms the HAP lattice decomposition.

The cytotoxicity determined by MTT assay indicates that HAP/Lig coating with 1 *wt* % Lig slightly reduced survival of healthy unstimulated PBMC. Therefore, the HAP/Lig coating is a promising non-toxic biomaterial for tissue engineering. However, the absence of zones of inhibition of bacterial growth around the tested HAP/Lig (1 *wt* % Lig) coatings indicates that lignin has no significant effect on the antibacterial properties of hydroxyapatite.

The morphology of Ag/HAP/Lig coating with 1 *wt* % Lig was homogenous without any fractures and the Ca/P ratio of 1.62 is similar to the stoichiometric value of hydroxyapatite (1.67). In our study immediate and continuous release of Ag^+^ ions indicates the optimal inhibitory concentration of antibactericidal agents that diminish the growth of bacteria strain *S. aureus* TL. Cytotoxicity testing of our composite material revealed that Ag/HAP/Lig coating with 1 *wt* % Lig can be classified as non-toxic against PBMC cell lines.

The newly formed bone-like plate-shaped apatite crystals observed on the surface of Ag/HAP/Lig coating after soaking for 7 days in SBF confirmed its bioactivity by SEM, XRD and ATR-FTIR results. Spontaneous growth on the surface of Ag/HAP/Lig coating of biologically active bone-like apatite layer can be tracked by impedance changes during soaking in SBF. However, this new apatite does not disturb the silver ion release from coating material and therefore the combination of the two results provides a strong platform for developing materials that are both bioactive and antimicrobial. Effective lignin concentration of 1 *wt* % provided for an excellent lead candidate for future biomedical application.
